# Exploring the Interplay Between Inflammatory and Metabolic Markers in Depression Treatment Outcomes: A Focus on Interleukin-6 and Ghrelin

**DOI:** 10.1192/bjo.2025.10162

**Published:** 2025-06-20

**Authors:** Jae-Min Kim

**Affiliations:** Chonnam National University Hospital, Gwangju, Korea, Republic of

## Abstract

**Aims:** This study aimed to elucidate the modulating effects of serum ghrelin on the relationships between interleukin-6 (IL-6) and antidepressant treatment outcomes, particularly focusing on 12-week remission and 24-month relapse in patients with depressive disorders.

**Methods:** We analysed baseline serum levels of ghrelin and IL-6 in 1,086 patients engaged in a naturalistic, stepwise antidepressant treatment protocol. Remission was assessed using the Hamilton Depression Rating Scale (HAMD), with a score of 7 or less defining remission at 12 weeks. Patients achieving a response (HAMD ≤14) at this interval were tracked for relapse (HAMD >14) quarterly up to 24 months. Logistic regression models, adjusting for sociodemographic and clinical variables, explored the interactive effects of these biomarkers on treatment outcomes.

**Results:** Our analysis indicated that while serum ghrelin levels did not directly impact treatment outcomes, they significantly modulated the relationship between high IL-6 levels and the likelihood of non-remission at 12 weeks as well as relapse at 24 months. Notably, elevated IL-6 was strongly associated with these negative outcomes primarily in the context of lower ghrelin levels. The modulatory effects of ghrelin were statistically significant in the context of relapse after controlling for relevant covariates.

**Conclusion:** The findings from this study underscore the critical interplay between inflammatory and metabolic markers in determining the trajectory of depression treatment outcomes. By demonstrating the significant roles of IL-6 and ghrelin, particularly their interactive effects, this research highlights the potential to enhance personalized antidepressant strategies through the integration of biomarker profiles. Future investigations should focus on unravelling the dynamic mechanisms behind these interactions, which could pave the way for refining prediction models for treatment responsiveness and developing targeted interventions that more effectively address the complexities of managing depression.

